# Isolation of primary human liver cells from normal and nonalcoholic steatohepatitis livers

**DOI:** 10.1016/j.xpro.2023.102391

**Published:** 2023-07-04

**Authors:** Xiao Liu, Kevin Lam, Huayi Zhao, Sadatsugu Sakane, Hyun Young Kim, Alvaro Eguileor, Karin Diggle, Shuai Wu, Raquel Carvalho Gontijo Weber, Pejman Soroosh, Mojgan Hosseini, Kristin Mekeel, David A. Brenner, Tatiana Kisseleva

**Affiliations:** 1 Department of Medicine, University of California, La Jolla, San Diego School of Medicine, La Jolla, CA, USA; 2 Department of Surgery, University of California, La Jolla, San Diego School of Medicine, La Jolla, CA, USA; 3 Department of Pathology, University of California, La Jolla, San Diego School of Medicine, La Jolla, CA, USA; 4 Janssen Pharmaceutical R&D, Immunometabolism Obesity and Metabolic Disorders, San Diego, CA, USA; 5 State Key Laboratory of Cellular Stress Biology, School of Pharmaceutical Sciences, Xiamen University, Xiamen, China

**Keywords:** Cell Biology, Health Sciences

## Abstract

Here, we present a protocol for isolating human hepatocytes and neural progenitor cells from normal and nonalcoholic steatohepatitis livers. We describe steps for perfusion for scaled-up liver cell isolation and optimization of chemical digestion to achieve maximal yield and cell viability. We then detail a liver cell cryopreservation and potential applications, such as the use of human liver cells as a tool to link experimental and translational research.

## Highlights

• Human liver cells change in response to NASH and development of liver fibrosis
• Human liver cells can be used to study signaling and disease-associated crosstalk
• Development of liver cell isolation techniques are critical for translational research

## Abstract (editor highlights)

Publisher’s note: Undertaking any experimental protocol requires adherence to local institutional guidelines for laboratory safety and ethics.

## Graphical abstract

**Figure:**
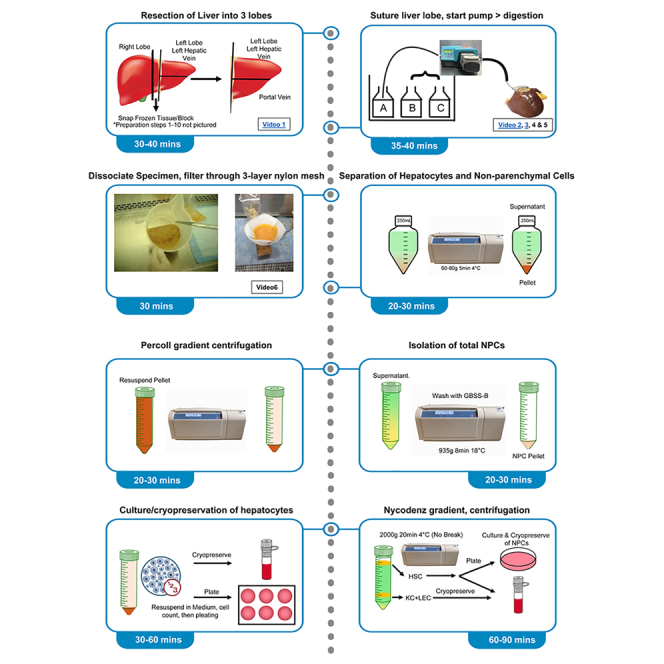

## Before you begin

**Figure 1:**
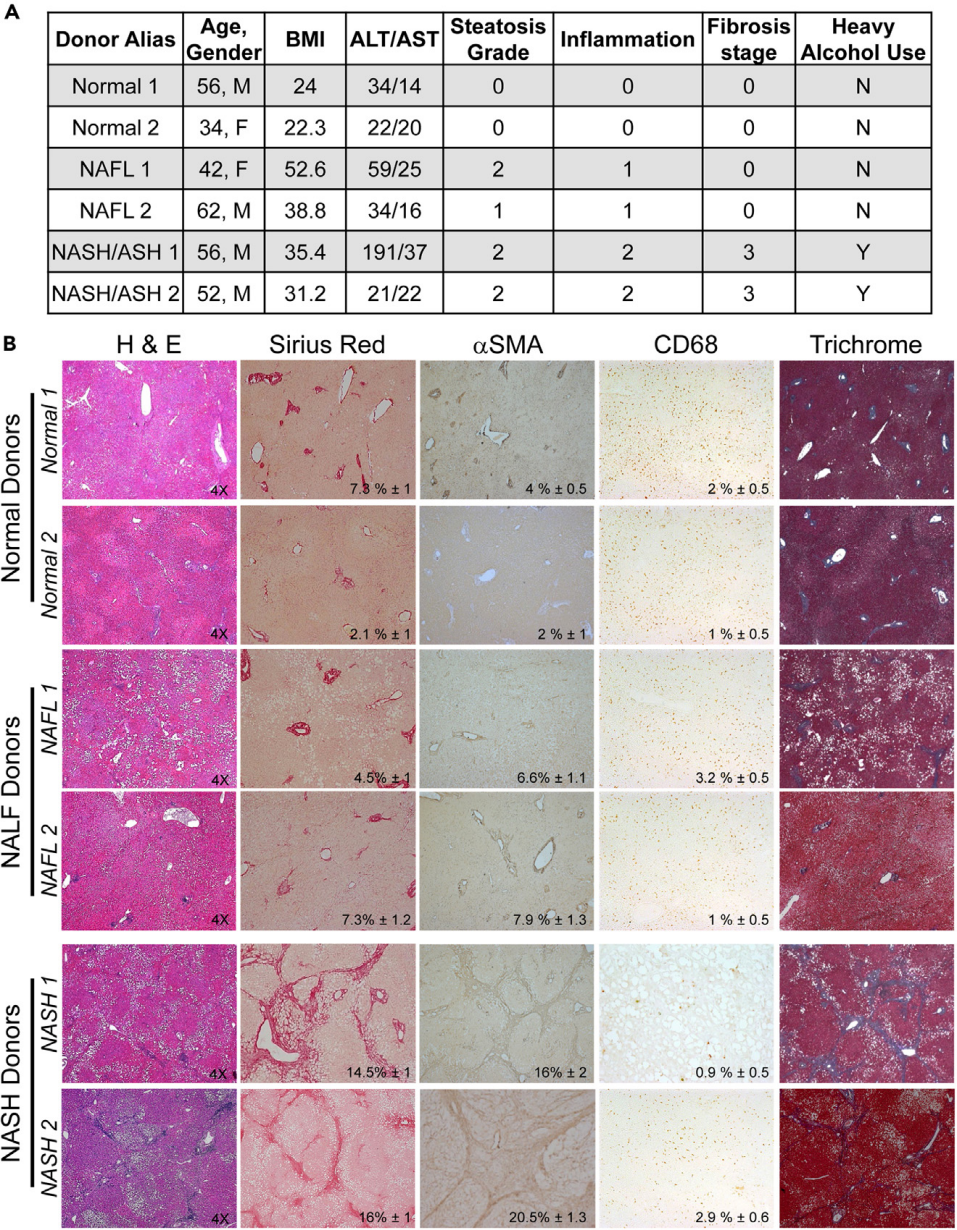
Characterization of Normal, NAFL, and NASH livers (A) Donor information: donors without underlying liver disease (Normal), donors with NAFL and donors with NASH were diagnosed based on liver function, histology, and history of alcohol use. Representative donor (n = 2/condition) information available from OPO is listed. (B) Human livers (Normal, n = 2, NAFL, n = 2 or NASH, n = 2) were stained for H&E, Sirius Red, αSMA, CD68, Trichrome, positive area was calculated as percent of total area using Image J, representative micrographs are shown (×4 objective), p < 0.5.

Institutional permissions: Deidentified livers declined for transplantation are obtained via Lifesharing Organ Procurement Organization (OPO) and used in this study. The patient consent was obtained by www.lifesharing.org. This project (171883XX) has been reviewed by the Director of the UCSD HRPP, IRB Chair, or IRB Chair’s designee and is certified as not qualifying as human subjects research according to the Code of Federal Regulations, Title 45, part 46 and UCSD Standard Operating Policies and Procedures, and therefore does not require IRB review. Livers were graded for steatosis, inflammation, and fibrosis by a pathologist using a double-blinded method and identified as normal and NASH (Table 1). Sex, gender, or age of human donors is not relevant for this protocol, as we obtain all livers that are offered by OPOs, which also provide de-identified donor’s consent and donor history **(**Figures 1A and 1B**).**

**Table 1:** NASC/CRN grading criteria

Grade	Criteria
Steatosis grade:	**0** < 5%; **1** - 5–33%; **2** - 34–66%; **3** > 66%
Steatosis distribution:	Centrilobular or diffuse
Lobular inflammation:	**0** - none; **1** < 2 foci/20× field; **2** - 2–4 foci/20× field; **3** > 4 foci/20× field
Hepatocellular ballooning:	**0** - none; **1** - few, **2** - many
Portal inflammation:	**0** - none; **1** - mild, **2** > mild, **3** - severe
Fibrosis (requires Trichrome stain):	**0** - none **1a** - mild zone 3 perisinusoidal fibrosis **1b** - moderate zone 3 perisinusoidal fibrosis **1c** - portal fibrosis only **2** - zone 3 perisinusoidal and periportal fibrosis **3** - bridging fibrosis **4** - cirrhosis

NASH diagnosis correlates with steatosis, related chronic steatohepatitis, and fibrosis. Liver sections (1 × 1 cm) graded > or = 5 is used as the cutoff for a diagnosis of NASH. Livers with scores of less than 3 are diagnosed as "not NASH"^21^.

### Experimental design

Primary cell isolation can be divided into two general steps: (i) Suturing of profusion tubing and digestion of liver tissue using EGTA, collagenase, protease, and/or pronase solutions; and (ii) density gradient–based separation of different cell populations. The underlying principle is that cell types are readily separated based on their different buoyancy and cell densities (Figure 3).

Proper perfusion of the liver lobes is the most crucial step for a successful cell isolation. Temperature and enzyme concentrations are key determinants of enzymatic activity, and they are highly relevant for successful hepatic digestion. As such, it is important to adjust the temperature of the water bath and/or the length of connecting tubes. The setup is adjusted to achieve a temperature of 39.5 °C at the end of the perfusion tube where solutions enter the blood vessels. It is crucial to prewarm the pronase, collagenase, and protease solutions to 39°C.

## Key resources table

**Table:** 

REAGENT or RESOURCE	SOURCE	IDENTIFIER
**Chemicals, peptides, and recombinant proteins**		

Pronase	Roche	10-165-921-001
Collagenase D	Roche	11-088-874-103
Collagenase MA	VitaCyte	001-2030
AOF BP Protease	VitaCyte	003-1000
DNase I	Roche	10-104-159-001
Potassium chloride (KCl)	Sigma-Aldrich	P9541-500G
Magnesium chloride hexahydrate (MgCl_2_.6H_2_O)	Sigma-Aldrich	M2670-100G
Magnesium sulfate heptahydrate (MgSO_4_.7H_2_O)	Sigma-Aldrich	M5921-500G
Sodium phosphate dibasic (Na_2_HPO_4_)	Sigma-Aldrich	S3264-500G
Potassium phosphate monobasic (KH_2_PO_4_)	Sigma-Aldrich	P9791-100G
D- (+)-Glucose	Sigma-Aldrich	G8270-1KG
Sodium bicarbonate (NaHCO_3_)	Fisher	S233-500G
Calcium chloride dihydrate (CaCl_2_.2H_2_O)	Fisher	C70500
Dexamethasone	MP Biomedicals	0219456180
Nycodenz AG	Accurate Chemical	AN1002424
HEPES	VWR	45000-690
EGTA	Fisher	50-255-957
Gey’s balanced salt solution B (GBSS/B)	Fisher	J67569-K2
10× DPBS	VWR	45000-426
Hanks’ balanced salt solution (HBSS)	Fisher	14175-095
Eagle’s Minimum Essential Medium (EMEM)	VWR	45000-386
PERCOLL	Sigma-Aldrich	GE17-0891-09
Fructose	VWR	97061-236
HMM SingleQuot Kit	Lonza	CC-4192
Cellbanker1	AMSBIO	11888
CS10	BioLife Solutions	210102
Surgical grade glue	VWR	37001-738
CD11b MicroBeads, Human and Mouse	Miltenyi Biotec	130-049-601
CD31 MicroBead, Human	Miltenyi Biotec	130-091-935
DMEM	Fisher	11965118
Williams’s E Medium, No Phenol Red	Fisher	A1217601
Antibiotic-antimycotic 100×	Gibco	15240062
Penicillin-streptomycin-glutamine 100×	Gibco	10378016
FBS	Gemini Bio-Products	100-106

**Other**		

Water Bath	Thermo	GP 10
Refrigerated Benchtop Centrifuge with swinging bucket rotor	Thermo	ST4R plus
CryoMed controlled-rate freezer	Thermo	TSCM34PV
Perfusion pump and tubing	Cole-Parmer	07525-40 / 96410-16
Biosafety cabinet	Thermo	10445753
Inverted microscope	OLYMPUS	CKX53
Cell culture incubators with 5% CO_2_, 37°C	Eppendorf	CellXpert C170
Glass door refrigerator	Thermo	FYC-335
−20°C freezer	Thermo	FDW-FL368
−80°C freezer	Thermo	905GP-ULTS
Precision balance	Sartorius	SQP
Cell counter/hemocytometer	MARIENFELD	065-0010
Pipet-aids	Drummond	XP2
Syringe 10 mL and 60 mL	Fisher	BD309604/BD309653
2-0 and 3-0 suture	ETHICON	685 / 632G
Sterile pipettes: 10 and 25 mL	JET BIOFIL	GSP-010-010 / GSP-010-025
Conical tubes: 15 and 50 mL	Corning	430790 / 430828
Conical tubes: 250 mL	Thermo	276814
Bottle top filter, 0.2 μm	JET BIOFIL	FNY202025
2 mL cryovials	Corning	CLS430488
Nylon mesh 500, 250 and 85 μm	CS Component Supply	U-CMN-500/250/85
Catheters, Luer adaptors (CP03800-08) and tubing	Cole-Parmer	96410-16 / 45504-26 / 45501-04
Surgical instruments scissor, 5″ straight blunt/blunt	ROBOZ	RS-6010
Microdissecting serrated forceps straight tip	ROBOZ	RS-8120
Microdissecting serrated forceps slight curve	ROBOZ	RS-5135
Micro dissecting forceps	ROBOZ	RS-5238
Hegar Baumgartner length 51/4"	ROBOZ	RS-7850
No.5 45 Deg Dumont Inox Tip Size .05 × .01 mm Biologie Tips	ROBOZ	RS-5005
Cole-Parmer Luer adapters, male slip Luer to 1/8″ ID, 25/Pk	Cole-Parmer	45504-26
Cole-Parmer Female Luer to 1/8″ hose barb	Cole-Parmer	45501-04
Masterflex Pump Tubing Silicone Tubing (Platinum) L/S 16 Length 25 Feet (7.6 m)	Masterflex	96410-16

∗This Percoll pH does not depend on the temperature.

∗All the surgical instruments, tubing and Nylon meshes need to sterilize before use.

## Materials and equipment

**Table:** Buffer A

Reagent	Final concentration	Amount
EGTA (0.5 M)	1.0 mM	1 mL
HBSS	N/A	500 mL
**Total**	**N/A**	**500 mL**

∗Prepared fresh under sterile conditions, 30 min before the start of the isolation procedure.

**Table:** Buffer B

Reagent	Final concentration	Amount
HEPES (1 M)	25 mM	12.5 mL
Collagenase MA	5000 U/mL	1 pack
AOF BP Protease	2200 U/mL	1 pack
EMEM	N/A	500 mL
**Total**	**N/A**	**500 mL**

∗Prepared fresh under sterile conditions, 30 min before the start of the isolation procedure.

**Table:** Buffer C

Reagent	Final concentration	Amount
HEPES (1 M)	25 mM	12.5 mL
Pronase	0.4 mg/mL	200 mg
EMEM	N/A	500 mL
**Total**	**N/A**	**500 mL**

∗Prepared fresh under sterile conditions, 30 min before the start of the isolation procedure.

**Table:** Buffer D

Reagent	Final concentration	Amount
HEPES (1 M)	25 mM	12.5 mL
Collagenase D	0.7–0.9 mg/mL	350–450 mg
EMEM	N/A	500 mL
**Total**	**N/A**	**500 mL**

Prepared fresh under sterile conditions, 30 min before the start of the isolation procedure.

**Table:** Gey’s balanced salt solution A, GBSS/A

Reagent	Final concentration	Amount
KCl	370 mg/L	370 mg
MgCl_2_.6H_2_O	210 mg/L	210 mg
MgSO_4_.7H_2_O	70 mg/L	70 mg
Na_2_HPO_4_	59.6 mg/L	59.6 mg
KH_2_PO_4_	30 mg/L	30 mg
Glucose	991 mg/L	991 mg
NaHCO3	227 mg/L	227 mg
CaCl_2_.2H_2_O	225 mg/L	225 mg
ddH_2_O	**N/A**	1 L
**Total**	**N/A**	**1 L**

∗Prepare the solution by dissolving the components listed in the table below in 1L of ddH2O. Adjust pH to 7.3–7.4 followed by 0.2 μm bottle top filter. Store at 4°C up to 6 months.

**Nycodenz I**: add 5.2 g–5.5 g Nycodenz in 15 mL GBSS/A.

∗Prepared fresh under sterile conditions.

**Nycodenz II**: add 3.63 g Nycodenz in 25 mL GBSS/A.

∗Prepared fresh under sterile conditions.

**DNase I (2 mg/mL)**: add 100 mg DNase I in 50 mL GBSS/B.

∗Aliquot 1 mL/tube, store −20°C up to 6 months, avoid freezing and thawing.

## Step-by-step method details

### Liver preparation before perfusion

**Timing: 30–40 min**

**Timing: 2–3 h (for step 18a)**

**Timing: 2–3 h (for step 18b)**

**Timing: 30 min for plating, 2–12 h for incubation, and 30 min for media change (for step 19a)**

**Timing: 30 min for plating, 2–3 weeks for cells to reach 90% confluence (for step 19b).**

**Timing: 1 h to prepare for control-rate step down with 1–2 h in freezer (for step 19c).**

**CRITICAL:** All liver tissues are obtained in accordance with federal, state, and institutional regulations. Once the donor liver is transferred into the lab, all procedures are then performed in a biological safety cabinet (Figures 2A–2C). PPE, shoes cover, gloves and bouffant cap are required.

1. Turn on biological safety cabinets and run for 10 min before using. Remove all materials and equipment from the cabinet. Clean the interior surfaces of the cabinet with an appropriate disinfectant, following the manufacturer’s instructions. Turn on biological safety cabinets and run for 10–30 min before using to purge any remaining contaminants from the air.

**Figure 2:**
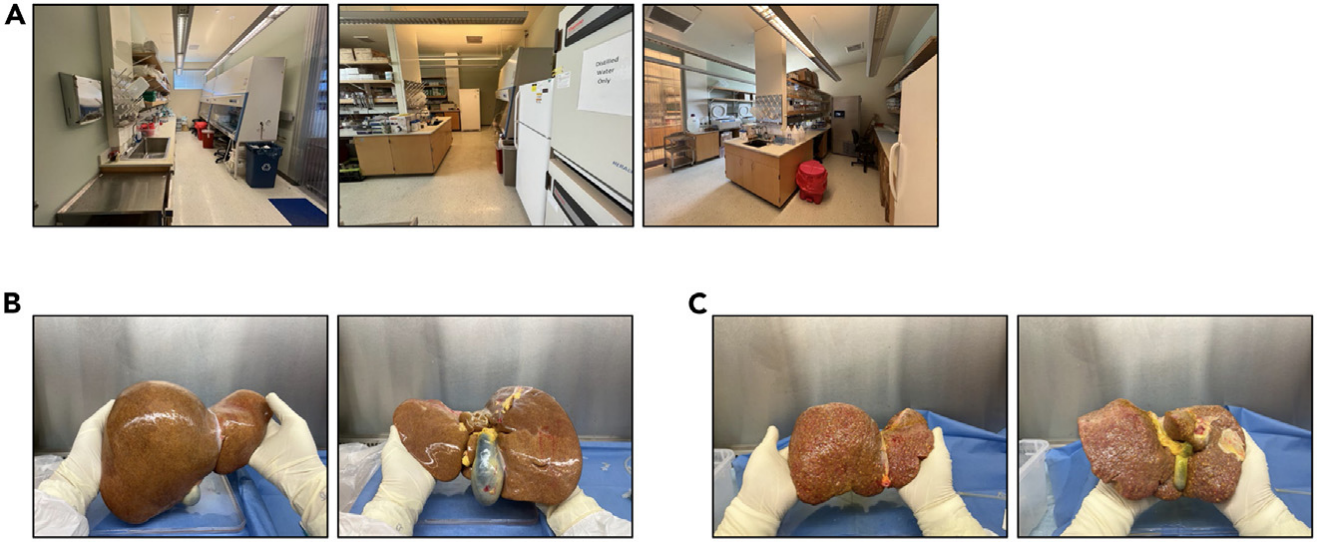
Isolation room images and workflow (A) Room images and workflow. Moving from left to right, the images depict the workflow of the cell isolation lab. The cell culture room (on the far right) is kept under the most strict and sterile condition to avoid possible contamination. (B and C) Images of normal (B) and cirrhotic (C) livers with the gallbladder attached.
2. Place peristaltic pump and filled water bath into the biological safety cabinet.
3. Turn on water bath and warm to 39°C.
4. Prepare perfusion solutions and place them in the water bath to warm up 20 min before starting the perfusion.

**CRITICAL:** The required volumes of solutions depend on the size of the liver. Dependent on the flow rate, the max flow rate used for 1 perfusion tubing/lobe is 30 mL/min. Only one tubing is usually used for perfusion of the left lobe at the perfusion rate of 30 mL/min for 20 min for max volume of 600 mL.

5. Load perfusion tubing into the peristaltic pump, with great care to maintain sterility, and place all ends into the Buffer A solution bottle.
6. Turn on the pump to prime the tubing and allow the solution to circulate continuously.
7. Cover a pan of ice with a plastic bag and place it into a second biological safety cabinet.
8. Place a sterile field over the ice pan and then place the liver on top of the sterile field.
9. Place a second sterile field next to the ice pan with all surgical instruments and needed supplies opened and placed on the sterile field aseptically.
10. Don sterile gloves, pick-up the liver and examine for any lacerations, bruising, and remarkable abnormalities.

**CRITICAL:** If gall bladder is still attached, use a scalpel to remove it (Figure 2B). Also, remove excess fat, muscle, and diaphragmatic tissues from the liver (Methods video S1).

**Figure 3:**
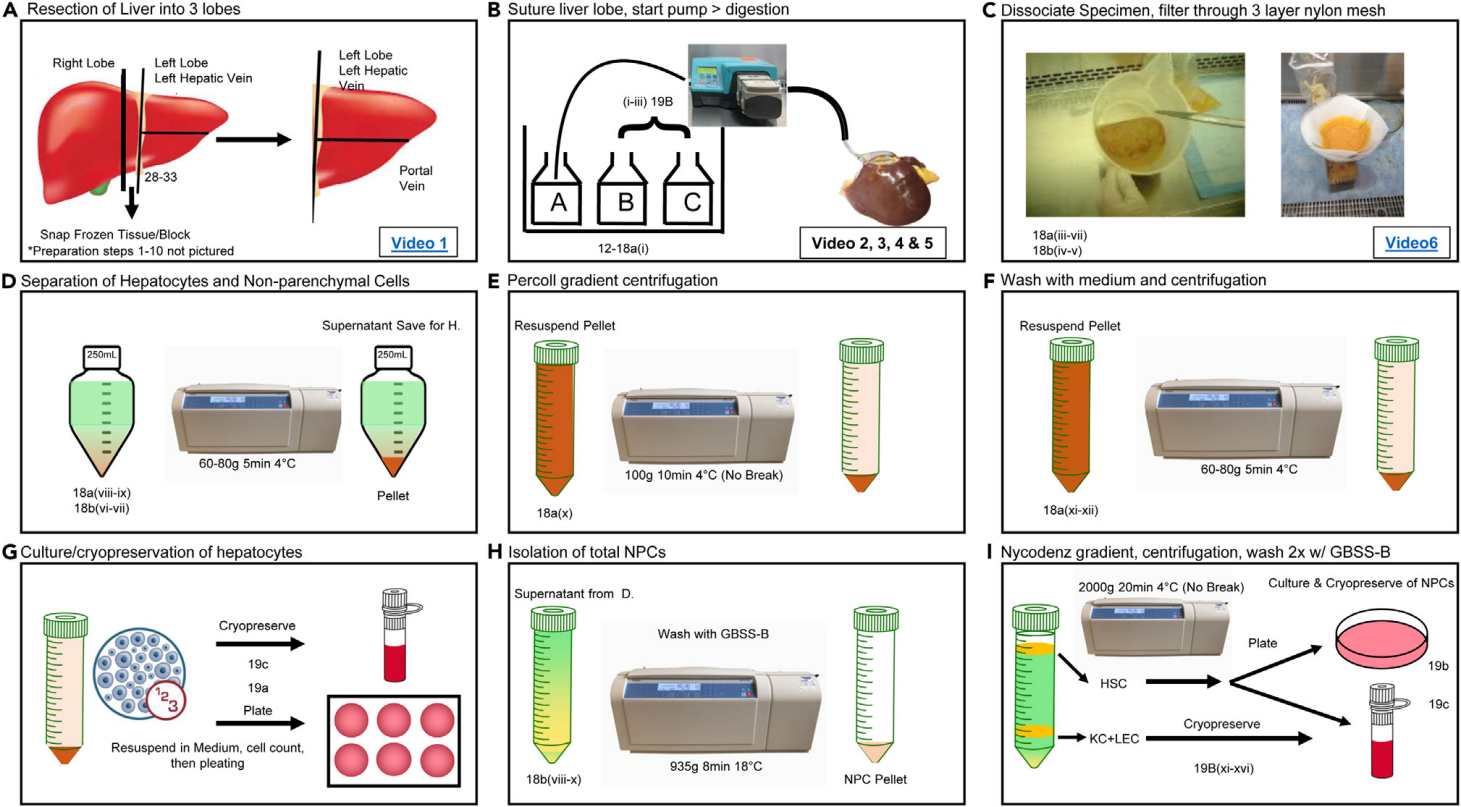
Overview of the human parenchymal and non-parenchymal isolation procedure (A) Resection of liver into 3 lobes (Methods video S1) and experimental setup. (B) Suturing the left and right lobes (Methods videos S2, S3, S4, and S5), connection to pump, sequential perfusion with buffer A containing EGTA, buffer B with pronase and buffer C with collagenase solutions. (C) The digested liver is dissociated and filtered through 3 layers of nylon mesh (Methods video S6). (D) Separation of hepatocytes and non-parenchymal cells. (E) Percoll gradient centrifugation for hepatocyte isolation. (F) Wash hepatocytes to prepare for cryopreservation. (G) Culture and cryopreservation of hepatocytes. (H) Cells are centrifuged, and the pellet is washed with GBSS-B buffer. (I) Nycodenz gradient centrifugation, the HSCs are collected by removing the cell layer from the gradient interface, washed then followed by cryopreservation or culture.

11. Resect the tissue into three pieces as shown in **(**Figure 3A**) (**Methods video S1).

**CRITICAL:** Use left lobe for hepatocyte isolation, use central lobe for snap frozen tissue and paraffin blocks, and use right lobe for non-parenchymal cell (hepatic stellate cell, Kupffer cell and endothelial cell) isolation.

12. Insert barbed catheters into the major portal and/or hepatic vessels but do not secure.
13. Connect a 60 mL syringe, filled with cold EMEM medium to the catheters, flush the liver tissue with EMEM medium to determine which vessels provide the most uniform perfusion of the liver, and to remove the residual blood (Methods video S2).

**CRITICAL:** Multiple vessels can be used but use as few vessels as possible while achieving maximal inflation of liver.

14. Secure the catheters into the selected vessels.
  ***Note:*** For this step, barbed luer adaptors can also be used to better secure the tubing in the vessel.
  a. Place 6–7 loose loop stiches through the blood vessel wall opening.
  b. Place the catheter (or luer adaptor) inside the vessel and tighten the suture around the blood vessel.
  c. Attach the tubing to the catheter.
  d. Place multiple loops of suture, tightening with a knot each time for the final securing of the perfusion tubing.
  e. Seal the cut surface of the liver tissue with sutures (size 2-0) or surgical grade glue.
    ***Note:*** The cut surface must be sealed to achieve full inflation of the liver (Methods videos S3 and S4)
    **CRITICAL:** All remaining major vessels on the cut surfaces must be closed with sutures or surgical grade glue.
15. Flush the liver tissue again using the syringe filled with cold EMEM to test for any major leaks from the liver.

**CRITICAL:** Close any leaks with glue or sutures (Methods videos S3 and S4).

16. Place liver tissue with catheters in a sterile plastic bag. Weigh the liver tissue with the bag closed.
17. Place the bag containing the tissue into the 39°C water bath **(**Methods video S5), connect to the peristaltic pump with flow rate 20–30 mL/min and perfuse the tissue with Buffer A for 10–15 min (Figure 3B).

**CRITICAL:** EGTA chelates calcium that leads to the separation of cell junctions and helps to remove any residual blood. Flow rate is based on the liver tissue size and number of catheters. Left lobe usually needs 500–600 mL of each perfusion buffer for complete digestion.

***Note:*** To ensure constant temperature during digestion, the liver lobe should be placed in a plastic bag and submerged in the water bath alongside the perfusion solutions (Figure 3B). A key step in the procedure is successful suturing and stabilization of the perfusion setup to avoid dislocation of the perfusion tubing during the perfusion (Figures 3A and 3B, and Methods videos S1, S2, and S3). Inflation of the whole liver lobe indicates successful insertion of tubing after beginning perfusion with buffer A

***Note:*** After perfusion with buffer A, vessels of the liver lobe should be a light brown color due to the EGTA chelating the remaining blood. This step helps to avoid contamination with red blood cells and clotting of hepatic vessels, which may result in incomplete perfusion and digestion. Aspirating buffer A from the bag before changing to the next buffer. Stop the pump while changing between buffers to prevent air. After perfusion with buffer B and/or C, the liver tissue starts fracturing under the capsule. The perfusion should be terminated after separation of liver tissue from the capsule is observed (Figure 3B).

18. Choose from the following options, depending on the type of cells to be isolated. Human hepatocytes are processed using option a. For non-parenchymal cells follow option b.
  a. Parenchymal cell isolation
    ***Note:*** For parenchymal cell isolation, it is important to calculate the proper Percoll concentration to be used depending on the pathology of the liver to ensure adequate quantities of live cells are collected (Figure 3D). When performing the density gradient centrifugation, it is important to proceed with the break off to avoid contamination of live cells with dead cells. While washing the parenchymal cell population, the cell pellet must be resuspended gently (Figures 3E–3G).
    i. Perfuse the liver tissue with Buffer B for 15–20 min.
      **CRITICAL:** Stop when liver tissue fractures and separates from the liver capsule.
    ii. Transfer the liver from the plastic bag into a sterile container containing pre-warmed (37°C) DMEM supplemented with 10% FBS, 25 mM HEPES, 100 nM Dexamethasone, 1× ITS (Insulin-Transferrin-Selenium), 1% Pen/Strep.
    iii. Dissociate digested liver tissue from the liver capsule with sterile scissors or by sterile gloved hand to release hepatocytes.
    iv. Filter cell suspension through sterile three-layer nylon mesh-covered funnels to remove cellular debris and clumps of undigested tissue.
      **CRITICAL:** Three-layer nylon mesh from inside to outside size 500 μm, 250 μm, 85 μm, and must be autoclaved before use. Repeat Step 18a(iv) as many times as needed to obtain the maximum number of cells (Figure 3C).
      ***Note:*** After the digestion, the liver is carefully removed and minced under sterile conditions (Figure 3C). The liver cell suspension is then filtered through a 3-layer nylon mesh 500, 250 and 85 μm (outside to inside) to separate undigested tissue remnants, and is washed with warm supplemented DMEM (Figure 3C and Methods video S6). The final step of the isolation procedure is a density gradient centrifugation including collection and counting of non-parenchymal and parenchymal cell types.
    v. Count cells using a hemocytometer and determine number of viable cells and yield with trypan blue staining.
    vi. Transfer cell solution to 225 mL conical tubes and top with warmed (37°C) DMEM supplemented with 10% FBS, 25 mM HEPES, 100 nM Dexamethasone, 1× ITS (Insulin-Transferrin-Selenium), 1% Pen/Strep.
      **CRITICAL:** A maximum of 1 billion hepatocytes should be added per 225 mL conical tube.
    vii. Perform low speed centrifugation at 60–80 *g* for 5 min at 4°C to isolate hepatocytes from other cell types in the suspension **(**Figure 3D).
    viii. While waiting during the centrifugation step, weigh the bag with catheters and subtract from the previous weight in step 16 to determine the true liver weight.
    ix. After centrifugation is complete, aspirate the supernatant leaving the hepatocyte pellets.
    x. Resuspend the pellets in the Percoll gradient solution, and centrifuge at 100 *g* for 10 min at 4°C!
      **CRITICAL:** Brake off! **(**Figure 3E**)**
      **CRITICAL:** When resuspending hepatocyte pellet, do not use a serological pipette. Resuspend the pellet gently by swirling the centrifuge tube with approximately 50–100 mL of media.
    xi. Following gradient centrifugation, aspirate the supernatant and wash hepatocyte pellet by gently resuspending in pre-warmed (37°C, 40 mL) DMEM supplemented with 10% FBS, 25 mM HEPES, 100 nM Dexamethasone, 1× ITS (Insulin-Transferrin-Selenium), 1% Pen/Strep. Plating conditions and cell density are described in the cell culture section.
    xii. Centrifuge 60–80 *g* for 5 min at 4°C **(**Figure 3F**).**
    xiii. Aspirate the supernatant and resuspend the pellet in a small volume of pre-warmed (37°C) DMEM supplemented with 10% FBS, 25 mM HEPES, 100 nM Dexamethasone, 1× ITS (Insulin-Transferrin-Selenium) 1% Pen/Strep.
    xiv. Check final viability of isolated hepatocytes and cell number. Hepatocytes can now be plated on Collagen pre-coated plates 18b. Alternatively, hepatocytes can be cryopreserved, see 19c **(**Figure 3G**).**
  b. Non-parenchymal cell isolation
    ***Note:*** For non-parenchymal cell isolation, after resuspending the liver cell pellet with density gradient medium, it is important to add the cell mixture very slowly on top of the Nycodenz gradient solution, and subsequently adding the density gradient-free medium on top (Figures 3H and 3I).
    Similarly, it is important to centrifuge the gradient with the brake off so that the Kupffer Cell+ liver sinusoidal endothelial cells (LSEC)-containing layer on the bottom gradient and the HSC-containing layer on top of the gradient are not disrupted (Figure 3I). After collecting HSCs and Kupffer Cell+LSEC fraction, cells are washed separately. Following final washes (Figures 3H and 3I), the purity of each fraction can be immediately assessed using trypan blue staining and calculated as percent of viable (trypan blue negative) vs. damaged (trypan blue positive) cells.^1^
    i. Perfuse the liver with Buffer C for 15–20 min.
    ii. Perfuse the liver with Buffer D for 15–20 min.
    iii. Stop the perfusion when the liver tissue begins to show fractures and separation from the liver capsule **(**Figure 3B**).**
      **CRITICAL:** Adjust digestion time to the point of full digestion of the liver tissue but not further.
    iv. Remove the liver tissue from the plastic bag and place in a sterile plastic beaker containing ice-cold EMEM. Then gently cut with sterile scissors to release cells.
    v. Filter the cell suspension through sterile three-layer nylon mesh-covered funnels to remove cellular debris and clumps of undigested tissues.
      **CRITICAL:** Three-layer nylon mesh from inside to outside size 500 μm, 250 μm, 85 μm, and must be autoclaved before use. Repeat this step as many times as needed to obtain the maximum number of cells (Figure 3C).
    vi. Transfer filtered NPC suspension into 50 mL conical tubes. Remove hepatocytes from the other cell types in the suspension by low-speed centrifugation at 60–80 *g* for 5 min at 4°C **(**Figure 3D**).**
      **CRITICAL:** Centrifugation speed depends on the fat content of the contaminating hepatocytes. Fattier liver needs higher centrifugation speed.
    vii. While waiting during the centrifugation step, weigh the bag with catheters and subtract from the previous weight in step 16 to determine the true liver weight.
    viii. Decant the supernatant and centrifuge at 935 *g* for 8 min at 18°C to collect total NPCs **(**Figure 3H**).**
    ix. Resuspend each pellet in 10 mL EMEM, and bring each tube up to 50 mL with EMEM. Spin again at 700 *g* for 8 min **(**Figure 3I**).**
    x. Discard supernatant, add 10 mL GBSS/B to resuspend the pellet, add 15 mL Nycodenz I, and bring the volume to 50 mL with GBSS/B. Mix well by inversion. This cell suspension will be used in the next step **(**Figure 3I**).**
      **CRITICAL:** Nycodenz I concentration must be adjusted based on the fat content in the liver. This is a critical step; the final concentration should be 10.4%–11%.
    xi. Layer 10 mL Nycodenz II on the bottom of 50 mL tube. Then, layer 30 mL cell mixture from step 18b(x). Finally, slowly layer another 10 mL of GBSS/B onto the cell mixture using a 20 mL syringe with 19-gauge needle held against the inside of the tube wall to avoid mixing the layers. Combine the remaining 20 mL of cell mixture from step 18b(x) to repeat the procedure in step 18b(xi). Centrifuge 2000 *g* for 20 min at 4°C **(**Figure 3I**).**
      **CRITICAL:** Centrifugation without brake is critical.
    xii. Under the top layer of clear GBSS/B solution is a white layer. This layer contains enriched hepatic stellate cells (HSCs). Collect this layer by carefully inserting a pipet through the clear GBSS/B layer, and transferring the HSC layer to new 50 mL tubes **(**Figure 3I**).**
    xiii. The second layer contains LEC and myeloid cells (Kupffer cells (KC) and bone-marrow derived myeloid cells). Remove enough of the remaining top solution to allow to safely collect the second layer with pipet. Collect this layer and transfer to new 50 mL tubes (for MACS selection). For myeloid cell purification, follow Miltenyi Biotec CD11b^+^ MicroBeads protocol. For LSEC purification, Miltenyi Biotec CD146+ Microbeads followed by Miltenyi Biotec CD31^+^ MicroBeads according to the vendor protocol.
    xiv. Add 40 mL of GBSS/B to wash cells and centrifuge 935 *g* for 8 min at 18°C
    xv. Repeat step 18b(xiv) but centrifuge 700 *g* for 8 min at 18°C
    xvi. Proceed to applications.
19. Proceed with option a to culture hepatocytes, option b to culture hepatic stellate cells, and option C to cryopreserve hepatocytes and hepatic stellate cells.
  a. Culturing hepatocytes
    i. Count cells using a hemocytometer and stain with trypan blue to determine the number of viable cells.
    ii. Plate hepatocytes (0.5 × 10^6^/mL) on Collagen coated plates and allow to attach for 2–12 h in a 37°C, 5% CO_2_ incubator.
    iii. Change media to pre-warmed (37°C) DMEM supplemented with 25 mM HEPES, 100 nM Dexamethasone, 1× ITS (Insulin-Transferrin-Selenium), 1% Pen/Strep.
    iv. Alternatively, if hepatocytes are used for cryopreservation as described in 19c, allow remaining hepatocyte stock to settle at 4°C for 1–2 h for cryopreservation.
  b. Culturing hepatic stellate cells (HSCs)
    i. Perform cell count.
    ii. Culture 0.5 × 10^6^ hepatic stellate cells with DMEM+10%FBS+1%AA in 10 cm dish.
    iii. Change media (DMEM+10%FBS+1%AA) every 3–4 days until cells are 90% confluent. At this point HSCs should be trypsinized, split, and further cultured, or cryopreserved.
  c. Cryopreservation of hepatocytes and hepatic stellate cells
    i. Count cells using a hemacytometer.
    ii. Resuspend cell pellet in appropriate freezing medium∗ based on cell type.
      **CRITICAL:** Hepatocyte Freezing Medium: CS10 + 100 mM Fructose. Hepatic stellate cell Freezing Medium: CELLBANKER® 1.
    iii. For Hepatocytes, aliquot 2 mL of cell suspension per freezing tube. Cell number per tube is 1.2 × 10^7^.
    iv. For hepatic stellate cells, aliquot 1 mL of cell suspension per freezing tube. Cell number per tube is 1.0 × 10^6^.
    v. Place tubes with cell suspension in Control-rate step down Freezer.
    vi. Start step down Program, and set the starting temperature to be 4°C.
      Step down Program

**Table:** 

Steps	Process	End temperature
1	Wait	at 4.0°C
2	1.0 °C/min S	to –4.0°C
3	25.0 °C/min C	to –40.0°C
4	10.0 °C/min C	to –12.0°C
5	1.0 °C/min C	to –40.0°C
6	10.0 °C/min C	to –90.0°C
7	End	

S = Sample Probe Temperature

C = Chamber Probe Temperature

C/m = Degrees Celsius per Minute

**Figure 4:**
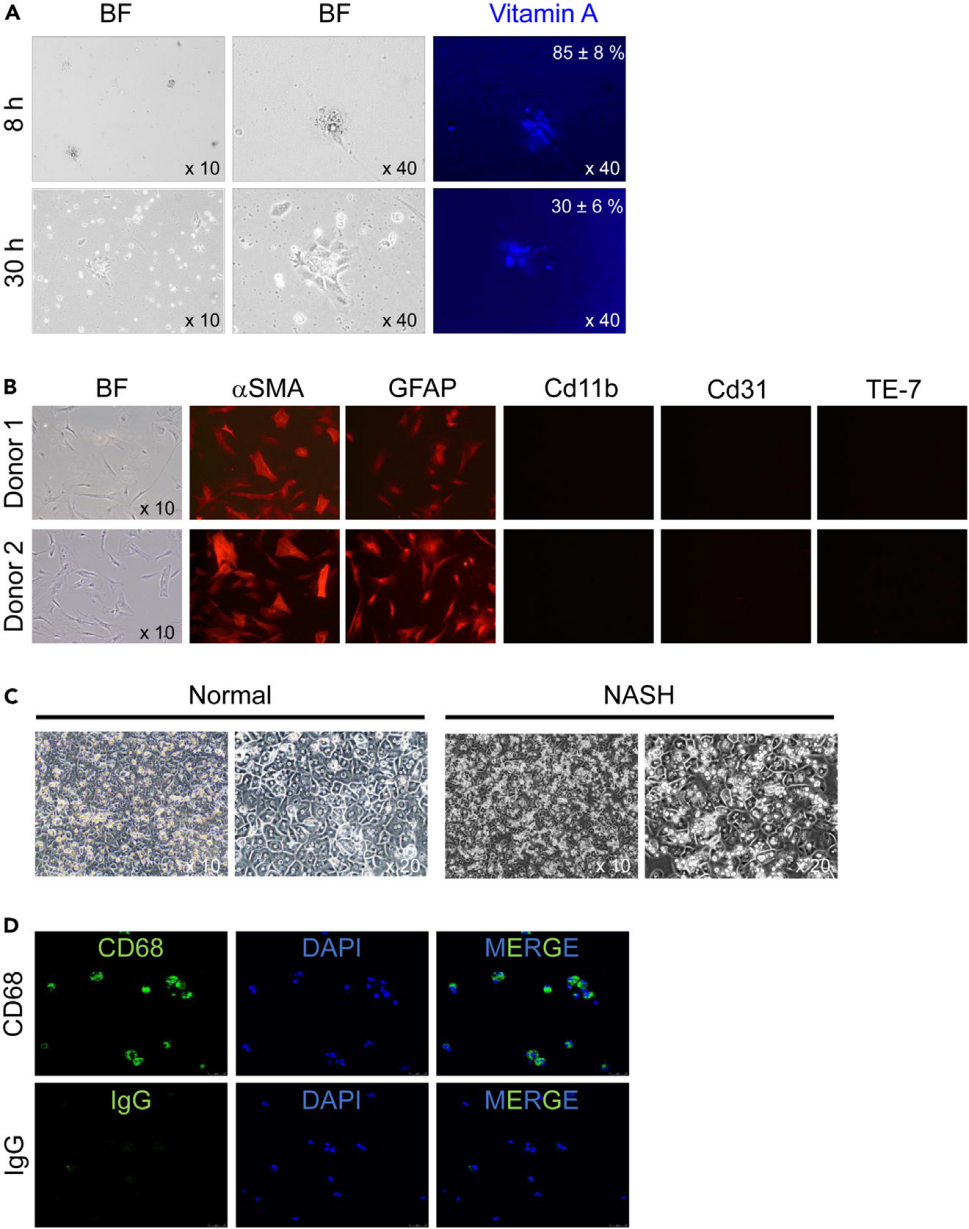
Characterization of human Hepatic Stellate Cells (A) Images of freshly isolated human HSCs cultured 8hs and 30hs. Phase-contrast (BF) and fluorescent images are shown. Expression of Vitamin A in lipid droplets was detected using UV light. Representative images are shown. (B) Primary human HSCs were immunostained for GFAP, αSMA, CD11b, CD31, and elastin (TE-7 Ab). Representative immunostating is shown for HSCs isolated from two donor livers. Expression of GFAP (>94%, p > 0.5) and αSMA was detected in HSCs. The HSC cultures were not contaminated with myeloid, endothelial cells or portal fibroblasts. Micrographs were taken x 10 objective. (C) Images of freshly isolated human hepatocytes cultured 6hs. Phase-contrast (BF) from normal and NASH liver. (D) Primary human Kupffer cells were immunostained for CD68, DAPI and IgG control.

This program is from the CryoMed controlled-rate freezer preset program 1, which is commonly used for 2.0 mL sample size according to the instruction of CryoMed controlled-rate freezer.
    vii. Transfer tubes into liquid nitrogen for long-term storage (vapor phase only).
      ***Note:*** HSCs can be identified by expression of Vitamin A (which is rapidly photobleached by the UV light) using fluorescent microscopy and immunocytochemistry for expression of GFAP and αSMA (Figure 4).^2^ qRT-PCR is another method which provides a sensitive assessment of contamination with specific cell types.

Methods video S1. Liver cutting_compressed, related to steps 10 and 11

Methods video S2. Flushing the liver, relatd to steps 12–18a(i), 18b(i–iii)

Methods video S3. Left Lobe Suturing_compressed, related to steps 12–18a(i), 18b(i–iii)

Methods video S4. Right Lobe Suturing_compressed, related to steps 12–18a(i), 18b(i–iii)

Methods video S5. Whole set up and prufussion steps, related to steps 12–18a(i), 18b(i–iii)

Methods video S6. Mincing of liver and Filtering, related to steps 12–18a(i), 18b(i–iii)

## Expected outcomes

### Validation of isolated human cells

#### Hepatocytes

Hepatocytes are identified by their characteristic morphology, multiple nuclei, and expression of Pan-CK, keratin 18 (K18), and HNF4α. Hepatocytes produce albumin, α-macroglobulin, transthyretin (TTR), cytochromes CYP4E3 (the marker of functional detoxification), which can be detected by qRT-PCR, enzymatic reactions, and Western blotting. Among the numerous enzymatic systems involved in hepatocyte metabolism, cytochromes P450 (such as CYP3E1) are upregulated in response to injury or stress. These cytochromes are monoxygenases mainly expressed in the liver.^3^ They oxidize a number of compounds and generate cytotoxic or genotoxic metabolites responsible for various conditions, such as AALD.^4^

#### Non-parenchymal fractions

**Figure 5:**
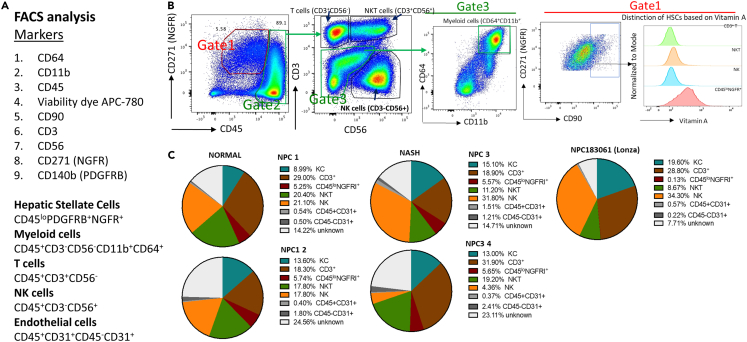
Analysis of isolated NPCs from normal and NASH livers Human NPCs from normal (n = 2) and NASH livers (n-2) were cryopreserved, then thawed and analyzed by flow cytometry using BD FACSymphony A1 cell analyzer according to the standard procedure. (A) Specific cell markers are listed. (B) The composition of cryopreserved human NPCs frpm normal livers (patients 1–2) and NASH livers (patients 3–4) were compared to commercially available NPCs (Lonza, NPC183061). Doublets and dead cells were excluded from the analysis. The frequency of myeloid cells (CD45^+^CD3^−^CD56^−^CD11b^+^CD64^+^), T cells (CD45^+^CD3^+^CD56^−^), NK cells (CD45^+^CD3^−^CD56^+^), and NKT cells (CD3+CD56+) is shown in Gates 2–3. The composition of Hepatic Stellate cells from Gate 1 (NGFR^+^VitaminA+) vs. NK, NKT, and T cells is shown (dot plots, and histograms). (C) The total NPC composition from normal and NASH livers (UCSD) and commercially available NPCs (Lonza) is shown in the pie charts. Cells that were not identified by the described markers were classified as unknown.

The composition of NPCs is best characterized using flow cytometry (Figures 5A and 5B). When the composition of non-parenchymal cells from normal and diseased donors was compared (vs. commercially available NPCs from Lonza Bioscience; https://bioscience.lonza.com/) similar cellular subsets were detected. As expected, the number of myeloid cells was increased in NPCs from the diseased donor, while the number of CD3^+^ T cells was decreased. Although LSECs were tested for expression of CD31, the percent of LSECs was low in both populations (Figure 5C). It is also recommended to test expression of CD146 in LSEC vs. all endothelial cells as expression of CD146 is associated with LSECs. CD146 and others such as Stabilin2 are good markers for LSECs. CD31 also detects endothelial cells from larger blood vessels, and these are not LSECs. Recent studies have suggested that Scavenger receptor type B1(SR-B1) is specifically expressed by LSECs.

HSCs: HSCs are identified by expression of NGFRI, and Desmin. Freshly isolated HSCs from normal livers can also be stained for GFAP using fluorescent microscopy (Figure 4). Differential analysis should be used to rule out contamination with CD31^+^ LSECs, CD68^+^ myeloid cells, elastin^+^ activated portal fibroblasts (aPFs) and other cell types^5^(Figure 4B). HSCs express Vitamin A, independent of whether they are isolated from normal or diseased livers (Figure 4A). However, following the isolation, expression of Vitamin A is downregulated in culture (starting at ≈9h after plating) and completely undetectable after 24 h in culture.^6^^,^^7^ Downregulation of retinoids in human HSCs (compared to mouse HSCs, which retain retinoids in culture) can be explained by the increased content of retinoic acid in rodent diet vs. humans. In addition, Lrat expression (mRNA or protein) is relatively low in human HSCs vs. mouse HSCs.^8^

### Advantages

Large quantities of human liver cells, including parenchymal and non-parenchymal fractions, can be isolated and used immediately or cryopreserved (see Applications). Moreover, non-parenchymal cells can be further fractionated (before or after cryopreservation, using cell sorting^9^ or MACS technology^10^^,^^11^).

Large quantities (billions) of parenchymal and non-parenchymal cells can be isolated from the whole liver, resulting in purification of large numbers of highly purified (>90%) and viable (>85%) cells.

Parenchymal (non-steatotic hepatocytes) and non-parenchymal fractions can be cryopreserved, and further analyzed/fractionated after thawing, the composition of viable cells can be determined using trypan blue staining.

Non-parenchymal fraction can be further fractionated using gradient centrifugation method that allows separation of Hepatic Stellate Cells (due to the presence of Vitamin A-expressing liposomes, Figure 4A) from a population enriched in myeloid cells and endothelial cells. Although the expression of Vitamin A is downregulated in activated HSCs, it is not totally suppressed (Figure 4A), and the amount of remaining Vitamin A^+^ lyposomes is sufficient for HSC isolation using gradient centrifugation from normal or diseased livers. HSCs can be cultured to passage one, analyzed for purity (Figure 4B) and cryopreserved, thawed and further propagated to passage 3–4. The enriched population of myeloid and endothelial cells can be further purified using MACS beads conjugated anti-CD146 Ab and/or anti-CD31 Ab, the antibodies that are routinely used for isolation of LSECs. Anti-CD11b Ab is used for separation of myeloid cells. Purified cellular fractions can be cultured using the cell-specific conditions that have been previously developed and are widely used to culture endothelial cells and myeloid cells.^12^

### Applications

The current protocol provides a comprehensive methodology for the isolation of human parenchymal and non-parenchymal cells from normal and diseased livers. It allows the study of single cell-based gene expression profiles, epigenetic regulatory mechanisms, proteomic and other omics technologies to identify the specific pathways activated in the pathogenesis of NAFL, NASH/AALD(Figure 4C) compared to normal livers. Isolated cells can be used to perform functional validation of the obtained targets and provide a useful tool to study human liver cells in a wide range of applications, including 2D culture activation, co-culture and functional interactions with other cell types, and 3D cultures of human liver spheroids and “livers in a dish”.

This protocol can be used for translational research and commercial liver cell isolation. Large quantities of highly purified cells can be used for drug screening, and/or drug validation. On a smaller scale, 2D and 3D *in vitro* experiments can be designed to compare cells from the same or mismatched (normal vs. diseased) patients, or paremchymal vs. non-parenchymal fractions.^13^ These experiments can demonstrate strong patient-specific reproducibility due to the high number of cells available, which can be cryopreserved for ongoing experiments and future use.

Given the high viability and purity of isolated cells, the responses of each cellular population to different stimuli can be studied *in vitro* using 2D cultures, co-cultures.^13^ Responses of hepatocytes, myeloid cells and HSCs isolated from well characterized normal, NAFL, NASH, and AALD livers can be compared. Injury specific phenotypes of the major liver populations can be revealed. Similar studies can be performed using LSECs or other cellular subsets.

The gene regulation (gene expression profile and epigenetic landscape) of freshly isolated normal and steatotic human hepatocytes, myeloid cells, and HSCs can be studied using RNA-Seq or ATAC-Seq, or similar techniques, which allow simultaneous access to the areas of open chromatin within the distal and proximal promoter/regulatory elements of specific genes, and interrogate their potential co-regulation.

The correlation between the gene expression in normal vs. metabolically injured hepatocytes and activated myeloid cells and HSCs/myofibroblasts can be established. The cross talk and signaling pathways between distinct cellular subsets can be outlined.

Freshly isolated, as well as cryopreserved non-parenchymal fraction and parenchymal fractions from normal and NASH/AALD patients can be used for generation of 3D human spheroids. The mismatch of normal vs. diseased parenchymal/non-parenchymal cells can provide a useful insight into the cell interaction and signaling within human spheroids. Human spheroids can become a useful tool for drug screening.

An alternative to 3D liver spheroids is generation of bioprinted organoids that function as “livers in a dish”.^13^^,^^14^ Due to the presence of biodegradable resin, liver organoids demonstrate improved liver cell growth and interaction. They remain viable up to 40 days, develop liver-like architecture, and can be used for induction of NASH. Upon exposure to the NASH cocktail, human liver organoids develop steatosis, inflammation, and fibrosis. 3D human organoids can be used for translational research.

## Limitations

The limitations discussed below are not specific to this particular human cell isolation protocol, but are applied to all perfusion/gradient centrifugation-based cell isolation techniques performed on the metabolically injured livers from patients and experimental models of NASH/AALD.

Enzymatic digestion of the whole human liver requires the perfusion of solutions into the liver under specific flow rate. Although unlikely, perfusion can cause undesired tissue damage and stretching, resulting in alterations of the gene expression profiles of isolated cells. This obstacle can be overcome by addition of the RNA polymerase inhibitor flavopiridol to the perfusion solution.^10^ Liver cells prepared with or without flavopiridol can be compared in a pilot experiment using qRT-PCR or RNA-Seq to determine the importance of transcription arrest on gene expression profile of specific cellular populations.

Normal hepatocytes can be cryopreserved and used for further experiments after thawing. In general, we do not recommend to cryopreserve steatotic hepatocytes. The fragility of steatotic hepatocytes makes them susceptible to freezing/thawing artifacts.^15^^,^^16^ Therefore, the viability of steatoic hepatocytes is low after thawing. Meanwhile, freshly isolated steatotic hepatocytes are viable and can be used for 2D cell cultures or generation of 3D spheroids or organoids (Norona, L.N.. et al., 2015,Cellular & Molecular Mechanisms of Toxicity, Gordon Research Seminar).

Prolonged culturing can cause plastic activation of hepatocytes leading to their epithelial-to-mesenchymal transition.^17^ Cell fate mapping experiments have demonstrated that hepatocytes do not give rise to myofibroblasts *in vivo* in response to fibrogenic liver injury.^17^^,^^18^^,^^19^ Generation of 3D spheroids may provide an alternative method to investigate hepatocyte responses to stimuli.

Isolation of HSCs from NASH/AALD livers may present difficulties due to contamination with steatotic hepatocytes.^8^ Although the perfusion conditions (with pronase/collagenase) are designed to lyse hepatocytes, some small number of fat-loaded hepatocytes with light buoyancy can still be found in the HSC fraction. Culturing of freshly isolated HSCs eliminates damaged hepatocyte contaminations within 3 days.

Prolonged culturing may cause HSC activation.^8^ If HSCs need to be amplified for the purpose of large-scale drug screening, it is recommended to grow freshly isolated HSCs until confluent, then harvest and store after passage 1. Unlike other reports^20^ (https://genome.ucsc.edu/ENCODE/protocols/cell/human/Stellate_Crawford_protocol.pdf), we do not recommend amplifying HSCs more than passage 4,^8^ as they change their gene expression profile.

## Troubleshooting

### Problem 1

Liver does not fully inflate. Possible reason: Leak;Perfusion tubing is inserted in a suboptimal location;Catheter is not tightly secured (steps 12, 15).

### Potential solution

• Push the catheter deeper into the vessel and resecure.
• Correct leaks by first suturing leaking larger and medium size vessels closed on the cut surface, and then apply surgical glue over sutures.
• Remove the catheter from the major portal and/or hepatic vessel and re-insert at a different vessel.
• Apply surgical glue around the catheter suture site or remove catheter, attach to perfusion tube luer adaptor, and re-suture into liver.

### Problem 2

Liver does not submerge in water bath. Possible reason: Density of liver is too low (step 17).

### Potential solution

Remove air from the sterile plastic bag by submerging bag with liver in the water bath by hand leaving the open end above water.

### Problem 3

Incomplete digestion. Possible reason: Enzymes (steps 18a(i),18b(iii)).

### Potential solution

• Re-check enzyme concentrations.
• Re-check the time perfusion solutions are placed in water bath - too little or too much time in the water bath could decrease enzyme activity.
• Re-check duration liver is perfused: fibrotic livers require additional digestion time.
• Ensure water bath is at 39°C when digestion begins, and liver is completely submerged (see troubleshooting guidance for step 17 above)

### Problem 4

Low viability. Possible reason: Filtering; Overloading centrifuge tubes; Disturbing pellet; Rough handling (step 18a(v)).

### Potential solution

• Make sure three-layer nylon mesh is arranged in the correct size order and filter slowly to prevent overflow of mixture with undigested tissue.
• Maximum of 1 billion total hepatocytes should be added per centrifuge tube used in hepatocyte wash.
• Handle centrifuge tubes gently before aspirating to make sure healthy pelleted cells are not resuspended into wash media and lost in aspirant.
• Resuspend hepatocyte pellets by swirling gently with media. Do not use serological pipette which will cause mechanical lysing.
• Add 1 wash cycle to remove dead hepatocytes.

### Problem 5

Low yield. Possible reason: Incomplete digestion (step 18a(v)).

### Potential solution

See troubleshooting guidance for steps 18a(i) and 18b(iii) above.

### Problem 6

Gradient is disrupted. Possible reason: Layering is performed with too much ejection force or too quickly (step 18b (x, xi)).

### Potential solution

Before beginning the layering process, coat the sides of the centrifuge tube with solution already inside. If layering is disrupted, pipette the layered material out, mix the solution inside the tube, and layer again.

### Problem 7

Low viability; Low yield; Low purity. Possible reason: Filtering; Rough handling during isolation; Incomplete digestion; Gradient concentration incorrect; Contaminations with fatty hepatocytes (step 19b(i)).

### Potential solution

• See troubleshooting guidance for step 18a(v) above.
• Avoid pipetting cells with too much force as mechanical stress injures cells.
• See troubleshooting guidance for step 18a(i) and 18b(iii) above.
• Make sure the correct quantity of Nicodenz was measured.
• Perform additional hepatocyte purification spins, step 18b(vi).
• Consider additional FACS-sorting procedure to purify HSCs further.

### Problem 8

Slow cell growth and low density. Possible reason: Quantity of cells plated was inaccurate or cells plated were smaller sized (step 19b(iii)).

### Potential solution

Ensure sufficient cells are plated; if cell density per plate is too low either due to lower quantity plated or smaller cell size, HSCs will not grow.

## Resource availability

### Lead contact

Xiao Liu, xil094@health.ucsd.edu.

### Materials availability

Not applicable.

### Data and code availability

Not applicable.

## References

[1] El Taghdouini A., Najimi M., Sancho-Bru P., Sokal E., van Grunsven L.A. (2015). In vitro reversion of activated primary human hepatic stellate cells. Fibrogenesis Tissue Repair.

[2] Wake K. (1980). Perisinusoidal stellate cells (fat-storing cells, interstitial cells, lipocytes), their related structure in and around the liver sinusoids, and vitamin A-storing cells in extrahepatic organs. Int. Rev. Cytol..

[3] Xu J., Ma H.Y., Liang S., Sun M., Karin G., Koyama Y., Hu R., Quehenberger O., Davidson N.O., Dennis E.A. (2017). The role of human cytochrome P450 2E1 in liver inflammation and fibrosis. Hepatol. Commun..

[4] Park G.R., Pichard L., Tinel M., Larroque C., Elston A., Domerque J., Dexionne B., Maurel P. (1994). What changes drug metabolism in critically ill patients? Two preliminary studies in isolated human hepatocytes. Anaesthesia.

[5] Iwaisako K., Jiang C., Zhang M., Cong M., Moore-Morris T.J., Park T.J., Liu X., Xu J., Wang P., Paik Y.H. (2014). Origin of myofibroblasts in the fibrotic liver in mice. Proc. Natl. Acad. Sci. USA.

[6] Kisseleva T., Cong M., Paik Y., Scholten D., Jiang C., Benner C., Iwaisako K., Moore-Morris T., Scott B., Tsukamoto H. (2012). Myofibroblasts revert to an inactive phenotype during regression of liver fibrosis. Proc. Natl. Acad. Sci. USA.

[7] Liu X., Xu J., Rosenthal S., Zhang L.J., McCubbin R., Meshgin N., Shang L., Koyama Y., Ma H.Y., Sharma S. (2020). Identification of lineage-specific transcription factors that prevent activation of hepatic stellate cells and promote fibrosis resolution. Gastroenterology.

[8] Liu X., Rosenthal S.B., Meshgin N., Baglieri J., Musallam S.G., Diggle K., Lam K., Wu R., Pan S.Q., Chen Y. (2020). Primary alcohol-activated human and mouse hepatic stellate cells share similarities in gene-expression profiles. Hepatol. Commun..

[9] Zhou Y., Adewale F., Kim S., Su Q., Glass D., Sleeman M.W., Murphy A.J., Cheng X. (2021). Five-in-One: simultaneous isolation of multiple major liver cell types from livers of normal and NASH mice. J. Cell Mol. Med..

[10] Troutman T.D., Bennett H., Sakai M., Seidman J.S., Heinz S., Glass C.K. (2021). Purification of mouse hepatic non-parenchymal cells or nuclei for use in ChIP-seq and other next-generation sequencing approaches. STAR Protoc..

[11] Zhai X., Wang W., Dou D., Ma Y., Gang D., Jiang Z., Shi B., Jin B. (2019). A novel technique to prepare a single cell suspension of isolated quiescent human hepatic stellate cells. Sci. Rep..

[12] Kisseleva T., Song L., Vorontchikhina M., Feirt N., Kitajewski J., Schindler C. (2006). NF-kappaB regulation of endothelial cell function during LPS-induced toxemia and cancer. J. Clin. Invest..

[13] Soret P.A., Magusto J., Housset C., Gautheron J. (2020). In vitro and in vivo models of non-alcoholic fatty liver disease: a critical appraisal. J. Clin. Med..

[14] Roskos K., Stuiver I., Pentoney S., and Presnell S. In: Chapter 24 - Bioprinting: An Industrial Perspective. 395-411. Yoo J., Atala A., editors. Essentials of 3D Biofabrication and Translation. Bioprinting: An Industrial Perspective. Academic Press: Elsevier; 2015.

[15] Terry C., Mitry R.R., Lehec S.C., Muiesan P., Rela M., Heaton N.D., Hughes R.D., Dhawan A. (2005). The effects of cryopreservation on human hepatocytes obtained from different sources of liver tissue. Cell Transplant..

[16] Terry C., Bailey M., Mitry R.R., Lehec S.C., Dhawan A., Hughes R.D. (2006). Analysis of the effects of cryopreservation on rat hepatocytes using SELDI-TOF mass spectrometry. Cell Transplant..

[17] Taura K., Miura K., Iwaisako K., Osterreicher C.H., Kodama Y., Penz-Osterreicher M., Brenner D.A. (2010). Hepatocytes do not undergo epithelial-mesenchymal transition in liver fibrosis in mice. Hepatology.

[18] Scholten D., Osterreicher C.H., Scholten A., Iwaisako K., Gu G., Brenner D.A., Kisseleva T. (2010). Genetic labeling does not detect epithelial-to-mesenchymal transition of cholangiocytes in liver fibrosis in mice. Gastroenterology.

[19] Chu A.S., Diaz R., Hui J.J., Yanger K., Zong Y., Alpini G., Stanger B.Z., Wells R.G. (2011). Lineage tracing demonstrates no evidence of cholangiocyte epithelial-to-mesenchymal transition in murine models of hepatic fibrosis. Hepatology.

[20] Eyden B. (2008). The myofibroblast: phenotypic characterization as a prerequisite to understanding its functions in translational medicine. J. Cell Mol. Med..

[21] Kleiner D.E., Brunt E.M., Van Natta M., Behling C., Contos M.J., Cummings O.W., Ferrell L.D., Liu Y.-C., Torbenson M.S., Unalp-Arida A., et al. Design and validation of a histological scoring system for nonalcoholic fatty liver disease. 10.1002/hep.20701 15915461

